# Post-Cesarean Section Abdominal Wall Endometrioma

**DOI:** 10.7759/cureus.10088

**Published:** 2020-08-27

**Authors:** Allen Mao, Hunaid N Rana, Brad Steffler, Suzy Figarola

**Affiliations:** 1 Radiology, University of South Alabama College of Medicine/University of South Alabama Health University Hospital, Mobile, USA; 2 Radiology, University of South Alabama Health University Hospital, Mobile, USA

**Keywords:** endometrioma, endometriosis, c-section, abdominal wall

## Abstract

A 30-year-old female with a history of multiple cesarean sections presents to the emergency department with several months of right lower quadrant abdominal pain only associated with her menstrual cycles. CT abdomen and pelvis with contrast was remarkable for an abdominal wall mass that likely represented an endometrioma, and she was subsequently discharged with pain medications and conservative treatment. However, three months later, she returned to the ED, because her pain was unbearable and refractory to medical management. Interventional radiology was consulted for percutaneous biopsy of the soft tissue mass located in her rectus abdominus muscle. Following the procedure, the patient was started on ORILISSA^®^ (elagolix), the first FDA-approved oral treatment for the management of severe pain associated with endometriosis. We highlight an interesting case of post-cesarean section abdominal wall endometrioma implantation and describe the patient’s clinical course and disease management. The radiographic features of the mass are described and proposed mechanisms for the development of an abdominal wall endometrioma following a C-section is discussed.

## Introduction

Endometriosis is estrogen-sensitive endometrial tissue, such as endometrial glands and stroma, that develops outside of the uterine cavity. A more localized form of endometriosis is an endometrioma or “chocolate cyst”, which classically occurs in women of reproductive age. The most common location for an endometrioma is the ovary, followed by the anterior/posterior cul-de-sac and posterior broad ligament [1]. Other rare sites of extrauterine involvement that have been reported include the gastrointestinal tract, respiratory tract, and abdominal wall. Although the most commonly accepted mechanism for the development of endometriomas is attributed to retrograde menstruation, an abdominal wall endometrioma is likely due to a combination of endocrine, immune, and inflammatory pathways. Of note, endometrial seeding of the abdominal wall can occur postoperatively after a C-section, which is enhanced by chronic inflammation and impaired immunity [2]. Due to the multifaceted and partially understood mechanism of abdominal wall endometrioma formation, the diagnosis can be delayed. Histological examination of specimens is often required for diagnostic confirmation. Depending on the severity of the presentation, patients can be treated with non-steroidal anti-inflammatory drugs (NSAIDs) for symptomatic relief or with biopsy and laparoscopic resection of implants for definitive diagnosis and treatment.

## Case presentation

A 30-year-old G4P3013 African American female with past medical history of morbid obesity, three cesarean sections, and bilateral tubal ligation presents to the emergency department with right lower quadrant abdominal pain, nausea, and vomiting. The patient reports that her pain has been periodically occurring over the last several months and interestingly is only associated with her menstrual cycles. The abdominal pain is cramping in nature without radiation and localized to her right lower abdomen. She states that she feels a “bulge and bump” in this region. On physical exam, there was tenderness to superficial palpation in the right periumbilical region, without rebound tenderness, guarding, or any other signs of acute abdomen, in addition to an absent Rovsing’s sign. Due to her large body habitus and abdominal pannus, the mass was unable to be palpated.

**Figure 1:**
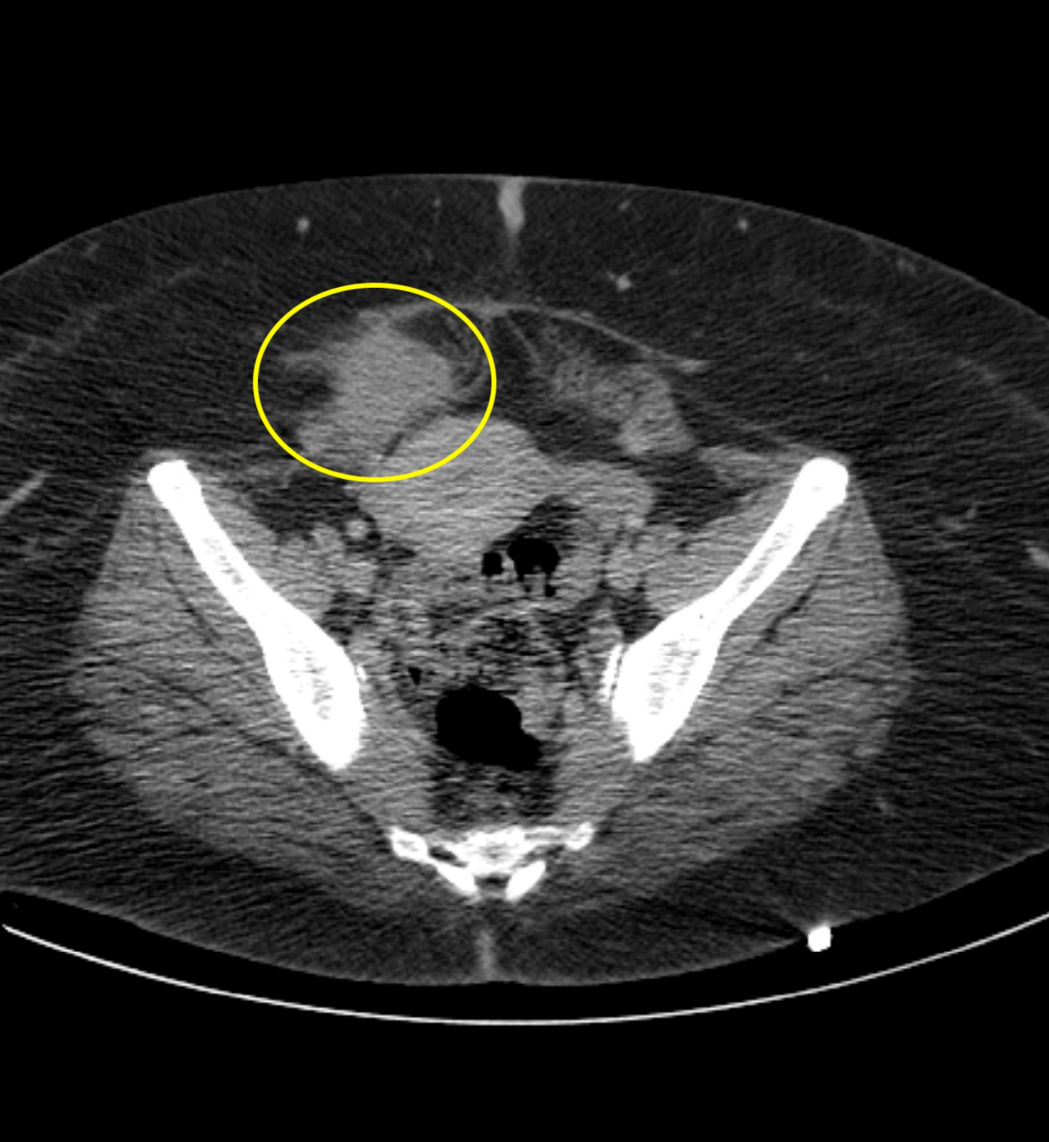
Initial emergency department CT axial demonstrates an ill-defined hyperattenuating soft tissue mass in the inferior right rectus abdominis muscle measuring approximately 5 x 4 cm with surrounding fat stranding and soft tissue edema that is consistent with an abdominal wall endometrioma (yellow circle).

**Figure 2:**
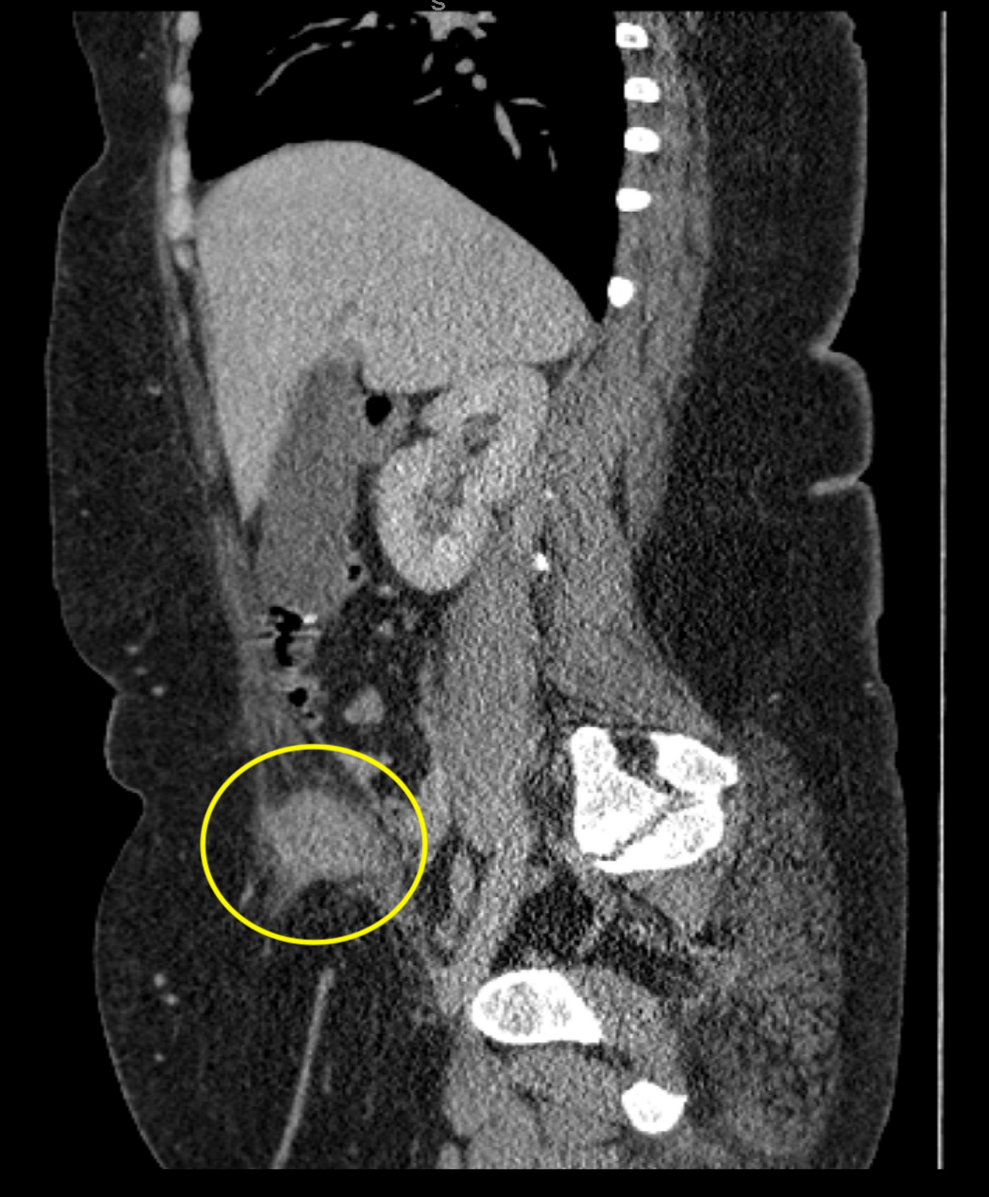
CT sagittal again exhibits an endometrioma contained in the right inferior rectus abdominus muscle, marked by an ill-defined hyperattenuating soft tissue mass (yellow circle).

The patient was given NSAID and anti-nausea medication, and a CT abdomen and pelvis with contrast was ordered to determine the etiology of the abdominal pain. CT showed an irregular, ill-defined 5 x 4 cm soft tissue mass in the inferior right rectus abdominus muscle at the right lower abdominal wall that was consistent with an endometrioma based on the patient’s past medical history (Figures 1, 2). After discussion with the emergency physician, recommendation was made for obstetrics and gynecology (OBGYN) to follow up the case regarding operative removal of the endometrioma and subsequent fascial repair. The patient was discharged with NSAIDs due to her hemodynamic stability.

After three months, the patient returned back to the ED with complaints of severe dysmenorrhea that was not alleviated with NSAIDs. OBGYN was consulted, but significant weight loss was recommended prior to surgical management due to the risk of wound healing complication and potential hernia development. Instead, interventional radiology (IR) was consulted for ultrasound-guided percutaneous biopsy of soft tissue mass in the rectus abdominus muscle. There were no postoperative complications on postprocedure ultrasound. Following the IR procedure, pathologic examination of three core biopsy soft tissue specimens confirmed the diagnosis of endometrioma due to the presence of ectopic endometrial stroma. The patient continued to follow-up with OBGYN and was placed on ORILISSA^®^ (elagolix), a gonadotropin-releasing hormone (GnRH) antagonist used to treat severe pain associated with endometriosis.

## Discussion

Endometriosis is defined as endometrial glands and stroma located at extrauterine sites that implant most commonly in the pelvis, such as the ovaries and fallopian tubes. These estrogen-sensitive ectopic implants can develop nearly anywhere in the body including infrequent locations such as the gastrointestinal tract, respiratory tract, or abdominal wall. A more localized form of endometriosis is an endometrioma or “chocolate cyst” that classically occurs in women of reproductive age. While the etiology and pathophysiology are not entirely understood, the most commonly accepted mechanism is due to retrograde menstruation, where endometrial cells flow in reverse through the fallopian tubes and into the pelvic cavity causing cyclical lower abdominal pain [1]. This produces proliferation and hemorrhage with each cycle that can lead to the manifestation of clinical symptoms, such as dysmenorrhea, dyspareunia, dyschezia, infertility, and cervical motion tenderness.

Interestingly, surgical operations, such as a C-section, can induce endometrial seeding of the abdominal wall, which is enhanced by chronic inflammation and impaired immunity. The development of an abdominal wall endometrioma likely is due to a combination of endocrine, immune, and inflammatory pathways rather than retrograde menses. Abdominal wall endometrioma is a relatively rare condition with a reported incidence of 0.03%-0.45% in women with previous C-section with a mean age at diagnosis of 35 years [3].

It is important to have a thorough review of a patient's prior surgical history when assessing an anterior abdominal wall mass that may be an endometrioma. When evaluating an abdominal mass, although much less likely, the differential diagnosis should include desmoid tumors, hematomas, and soft tissue sarcomas [3,4]. In our case, the constellation of chronic pelvic pain, abdominal wall mass on imaging, and history of C-section is highly suggestive of the diagnosis of an abdominal wall endometrioma.

Patients may initially present with chronic pain that is cyclical in nature with the menstrual cycle at the cesarean scar or incision site of the abdominal wall. Occasionally, an endometrioma located superficially under the skin may be visualized and appear blue, purple, or brown [5]. Ultrasound, CT, and MRI can all be used to characterize the mass. Due to low cost and easy accessibility, ultrasound is the recommended imaging modality to start diagnostic workup. On ultrasound, an endometrioma should appear hypoechoic, solid, and display vascularity [6].

CT commonly demonstrates non-specific findings of a solid, enhancing mass. Compared to CT, MRI is often preferred to suggest the diagnosis of endometrioma due to enhanced definition of soft tissues. There will be hyperintense, heterogeneous signal intensity of the affected area, often with high internal punctate signal intensity on both T1 and T2-weighted images, indicative of hemorrhage from the endometrial glands [7]. Another diagnostic clue may be the presence of a dark spot sign on T2-weighted sequences, which is an indicator of chronic hemorrhage [8]. Furthermore, percutaneous needle biopsy can be performed for diagnostic confirmation, often with IR guidance, in order to obtain pathologic tissue specimens with high accuracy. The definitive treatment of an abdominal wall endometrioma is via wide local excision with negative margins, which has a low recurrence rate. For patients who desire non-invasive medical management or are not surgical candidates, ORILISSA (elagolix) is a novel FDA-approved medication used to treat severe pain associated with endometriosis. This oral non-hormonal drug works by antagonizing GnRH receptors in the pituitary gland that leads to a dose-related reduction in estrogen production, the driving factor in endometriomas [9].

## Conclusions

We highlight an interesting case of an abdominal wall endometrioma status post C-section. The diagnosis requires heavily on a combination of history, presentation, and imaging for confirmation. This case emphasizes the need to have a high index of clinical suspicion when evaluating women with a prior surgical history of C-section who present with cyclical pain and an abdominal wall mass on imaging. Definitive treatment is via operative resection; however, oral medications are preferred for patients with certain comorbidities where surgery is unfavorable.

## References

[1] Koninckx PR, Ussia A, Adamyan L, Wattiez A, Gomel V, Martin DC (2019). Pathogenesis of endometriosis: the genetic/epigenetic theory. Fertil Steril.

[2] Carriero C, Dellino M, Capursi T, Cormio G (2017). Endometrioma of the abdominal wall after caesarean section. Open J Obstet Gynecol.

[3] Col C, Yilmaz EE (2014). Cesarean scar endometrioma: case series. World J Clin Cases.

[4] Saliba C, Jaafoury H, El Hajj M, Nicolas G, Haidar Ahmad H (2019). Abdominal wall endometriosis: a case report. Cureus.

[5] Goker A, Sarsmaz K, Pekindil G, Kandiloglu AR, Kuscu NK (2014). Rectus abdominis muscle endometriosis. J Coll Physicians Surg Pak.

[6] Francica G, Scarano F, Scotti L, Angelone G, Giardiello C (2009). Endometriomas in the region of a scar from cesarean section: sonographic appearance and clinical presentation vary with the size of the lesion. J Clin Ultrasound.

[7] Kocher M, Hardie A, Schaefer A, McLaren T, Kovacs M (2017). Cesarean-section scar endometrioma: a case report and review of the literature. J Radiol Case Rep.

[8] Corwin MT, Gerscovich EO, Lamba R, Wilson M, McGahan JP (2014). Differentiation of ovarian endometriomas from hemorrhagic cysts at MR imaging: utility of the T2 dark spot sign. Radiology.

[9] Taylor HS, Giudice LC, Lessey BA (2017). Treatment of endometriosis-associated pain with Elagolix, an oral GnRH antagonist. N Engl J Med.

